# Effect of Strongly Alkaline Electrolyzed Water on Silk Degumming and the Physical Properties of the Fibroin Fiber

**DOI:** 10.1371/journal.pone.0065654

**Published:** 2013-06-18

**Authors:** Ting-Ting Cao, Yuan-Jing Wang, Yu-Qing Zhang

**Affiliations:** Silk Biotechnology Laboratory, School of Biology and Basic Medical Sciences, Soochow University, National Engineering Laboratory for Modern Silk, Soochow University, Suzhou, P.R. China; University of Akron, United States of America

## Abstract

Strongly alkaline electrolyzed water (SAEW) was prepared by electrolysis of tap water in a laboratory-made water electrolyzer. The pH of stored SAEW was stable for more than one month. The hardness of the electrolyzed water was 30% lower and the Na^+^ concentration was 18% higher than those of the tap water. Silkworm cocoon shells were boiled in pH 11.50 SAEW at a ratio of 1∶40∼80 (W/V) for 20 min and the sericin layers around the silk fibroin fibers were removed completely. The tensile properties and thermal decomposition temperature of a single filament of silk fibroin obtained by the SAEW method were almost the same as those for the fiber obtained by the neutral soap, and much higher than those for the fiber obtained by Na_2_CO_3_ degumming. The results demonstrate that SAEW is an environmentally friendly and pollution-free silk degumming agent that allows highly efficient, low cost recovery of sericin.

## Introduction

Silk, a natural polymer of protein fibers produced by the silkworm *Bombyx mori*, is composed of 65∼75% fibroin, 20∼30% sericin and the remainder (∼5%) is composed of wax, pigments, sugars and other impurities. The silkworm cocoon is composed of fibroin, a crystalline protein fiber, surrounded by layers of the protein sericin, which reinforces the cocoon structure. The production of silk textile fibers or biomedical materials involves removal of the sericin that surrounds the natural fibroin fibers. Sericin contains 18 different amino acids; most are large molecular side chain, polar and hydrophilic amino acid residues and, therefore, sericin has the characteristics of glycerol-like absorption or release of moisture and protection against UV irradiation. The glue protein sericin is synthesized and excreted in the middle silkgland cells and can have markedly different degrees of solubility. The layer of sericin immediately surrounding the fibroin fiber is poorly soluble in water and is excreted in the posterior of the middle silkgland. The sericin surrounding this inner layer is water-soluble and is excreted in the anterior of the middle silkgland [1].

Sericin swells and dissolves in hot water, especially in alkaline hot water. Ancient Chinese people used hot water to cook the cocoons and then wound the silk onto a reel [2]. This raw silk was degummed or scoured by heating in alkaline aqueous solution. The use of alkalis as degumming agents is still essential for modern silk production processes, such as degumming or scouring, spinning and the production of medical biomaterials. Industrial silk production generates a huge amount of alkaline waste water containing sericin, which is difficult to recover, resulting in serious environmental pollution and enormous waste of biological resources [3], [4].

The methods used for silk degumming in the laboratory involve treatment with highly concentrated urea [5], or pH neutral soap (Marseilles soap) [6], [7], or Na_2_CO_3_. Boiling for 30 min in 0.2∼0.5% (W/V) Na_2_CO_3_ is the degumming method most commonly used in the laboratory. During this process, sericin is mostly hydrolyzed into peptides and free amino acids, which are difficult to separate and purify. A large number of alkaline substances are used for degumming; e.g. Bleach (NaClO) and softening agents are added to raw silk and silk fabrics for scouring and for the manufacture of spun silk as well as silk floss and silk biomaterials, making it difficult to separate and recycle the sericin present in vast amounts of degumming waste. Degumming at high temperature under high pressure [8] is a low-cost technique but it removes only the outer layer of sericin; the inner layer of sericin in contact with the fibroin fibers is not removed. Enzymatic degumming [9], [10] can completely remove the sericin by hydrolysis, but the degree of degumming efficiency is so low that it is unsuitable for large-scale industrial production. There are reports of the use of organic acids as silk degumming solvents [11], [12], [13]. Only a very small amount of the sericin materials released by degumming cocoon shells is utilized commercially. The sericin-derived material produced during degumming at high temperature under high pressure or by enzymatic degumming consists largely of sericin peptides. Typically, a preparation of >30 kDa products of silk sericin is used as a surface modifier for textile fibers and industrial products and there are reports of sericin peptides of <30 kDa, particularly 10 kDa, being used as a tyrosinase inhibitor [14], an antioxidant [15], an antidiabetic agent [16] and an antitumor factor [17], [18]. Sericin is a chemically inert non-ionic surfactant [19], [20] that does not induce pH changes, so it is suitable for mixing with other materials to produce sericin-based surfactants that are very gentle on human skin and particularly effective for the removal of oils. Sericin has long been used as an ingredient in cosmetics in Japan and other countries. Highly purified sericin can be used as a replacement for albumin or serum, thereby avoiding the introduction of animal pathogens [21], [22], [23]. Sericin is available commercially as a cell culture matrix or additive (Wako Product, Pure Sericin TM, Japan). Sericin alone or mixed with silk fibroin or other polymer material can be made into 3D porous human tissue engineering or biomimetic materials that can be used as a matrix for stem cell proliferation as well as for human tissue or organ regeneration [24]. It is likely that research and development of this silk protein for the production of medical tissue engineering materials will remain a focus of attention in the near future. Therefore, it is extremely important to develop novel environmentally friendly silk degumming agents for the recovery and utilization of sericin and/or sericin-derived materials.

A decade ago, Kitagawa reported that acidic or alkaline electrolyzed water can be used for degumming *B. mori* cocoon silk [25]. Alkaline (pH 11∼12) electrolyzed water was prepared by adding 0.107% electrolyzed accelerator (i.e. electrolytes such as sodium chloride, potassium carbonate etc.) to water. Seo in South Korea made a similar patent application in 2001 [26]. Later, Kim et al. used 0.2% (W/V) sodium carbonate as the electrolyte to prepare pH 11.6 alkaline electrolyzed water for silk degumming and sericin recovery [27]. The pH of the alkaline electrolyzed water reported above is stable for 8 days when stored at 4°C. It is generally believed that exposure to air, light, stirring and vibration during the storage of strongly alkaline or acidic electrolyzed water will affect the stability of the pH value, which tends to neutral within a few days. Hasegawa et al. added crystalline clay mineral salts as an electrolyte into water and the resulting pH 12.0 alkaline electrolyzed water was used for the degumming of modified silk fiber and fabrics and sericin recycling [28]. During the preparation of electrolyzed water described above, the electrolysis accelerator must be added for the preparation of strongly alkaline electrolyzed water (SAEW). An increased mineral salt content in the degumming solution affects the efficiency of sericin purification and recovery. Until now, apart from the patent applications mentioned above, there is no report of the use of SAEW as a degumming/scouring agent for silk floss, silk spinning or the production of raw silk fabrics or effects on the mechanical properties of the fiber.

## Results and Analysis

### Preparation and Storage Stability of SAEW

In this experiment, 28 L of tap water was electrolyzed to generate 18 L of acidic water (pH 3.00) and 10 L of pH 11.50 SAEW. The SAEW was filtered through a 0.45 µm paper filter to obtain a white precipitate that was dried to yield 0.7995 g of white powder. After electrolysis, the powder consists of salts such as calcium carbonate that are insoluble or poorly soluble in SAEW. This is equivalent to 0.7995 g of ions present in 28 L of tap water precipitated in SAEW; 28.55 mg/L salt ions were precipitated from tap water by electrolysis. We observed that the acidic electrolyzed water and the filtered SAEW were very transparent.

**Figure 1:**
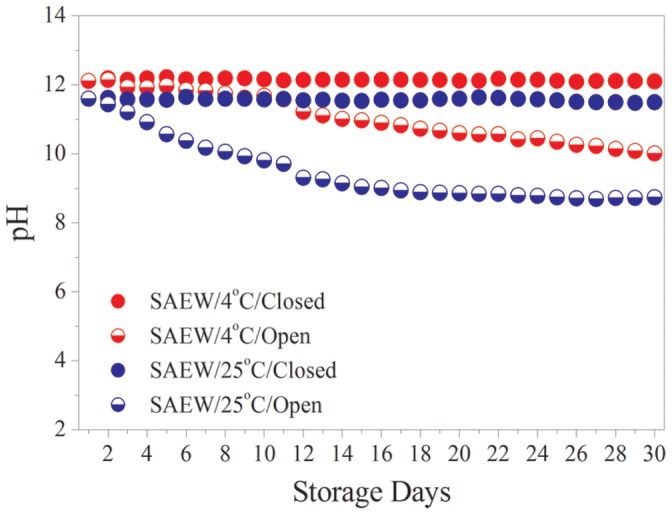
pH storage stability of SAEW.

In order to determine the pH stability of the electrolyzed water during storage, tap water was used to prepare pH 12.10 SAEW and pH 11.60 SAEW. Figure 1 shows that the pH 12.10 (red filled dots) and pH 11.60 (blue filled dots) SAEWs stored in closed containers at 4°C and at 25°C maintained their original pH value for 1 month, when the values were 12.00 and 11.50, respectively. When the two SAEWs were stored in open containers at 4°C and 25°C, their pH values decreased markedly; after 1 month the pH values were 10.81 and 8.22, respectively. It is clear that the stability of the SAEW pH value in closed containers is much greater than that in open containers at 4°C and at 25°C. The pH value of the SAEW stored in the open state would slowly decrease, because CO_2_ existed in the air would reacted with a higher concentrations of OH^−^ in the SAEW, resulting to generate a weak acid HCO_3_. As long as air is excluded, SAEW can be stored for long periods with a little change of pH. This result is a little inconsistent with the earlier report by Hasegawa et al [28] because of the addition of the electrolyte such as mineral salts, NaCl promoting water hydrolysis. The results presented above show that, under airtight storage conditions, the pH of SAEW is as stable as that of acidic electrolyzed water.

### Hardness of SAEW

Four types of water were analyzed: (1) pH 11.50 SAEW and (2) pH 3.00 acidic electrolyzed water were prepared with our laboratory-made water electrolyzer; (3) tap water (pH 8.00) and (4) ultrapure water (18.0 MΩ cm). Ca^2+^ and Mg^2+^, the main determinants of water hardness, as well as Na^+^ and K^+^ were measured (Table 1). The analysis gave the following results: tap water, pH ∼8, Ca^2+^ and Mg^2+^ together, 29.31 mg/L, Na^+^44.6 mg/L and K^+^5.05 mg/L. Ultrapure water, pH 8.23, Ca^2+^4.28 mg/L, Mg^2+^0.80 mg/L, Na^+^2.77 mg/L and K^+^0.91 mg/L. The concentrations of Ca^2+^, Na^+^ and K^+^ were greatly decreased in acidic electrolyzed water but the concentration of Mg^2+^ was little changed. The concentrations of Ca^2+^ (16.76 mg/L) and Mg^2+^ (3.47 mg/L) in pH 11.50 SAEW were decreased slightly, giving a total hardness of 20.23 mg/L, which is ∼70% of the value for the tap water. The concentration of Na^+^ was increased significantly to 52.96 mg/L, ∼18% higher than that of the tap water (44.60 mg/L), whereas the concentration of K^+^ (4.96 mg/L) was slightly lower than that of tap water. By the use of water electrolysis, we obtained pH 11.50 SAEW with water hardness reduced by 30% and a Na^+^ concentration ∼18% higher than that of tap water; therefore, the strong alkalinity of SAEW is mainly due to the high concentration of OH^–^ rather than a significant increase in alkaline ions resulting from water electrolysis.

**Table 1:** The alkaline ions and content in various water (mg/L).

	Detected elements	Ca^2+^	Mg^2+^	K^+^	Na^+^
Water	Wavelength (nm)	422.67	285.21	766.49	589.59
Ordinary tap water	pH 8.00	24.51	4.90	5.04	44.60
Ultrapure water	pH 8.23	4.28	0.80	0.91	2.77
Acidic electrolyzed water	pH 3.00	14.57	3.03	1.69	20.47
SAEW	pH 11.50	16.76	3.47	4.96	52.96

Note: water quality measurement by a Varian Vista MPX ICP spectrometer; ultrapure water (18.25 MΩ.cm) by an UP-II-10T ultrapure water machine. The values in the table are an average of three repeated measurements.

### Effect of pH on Silk Degumming Rate

**Figure 2:**
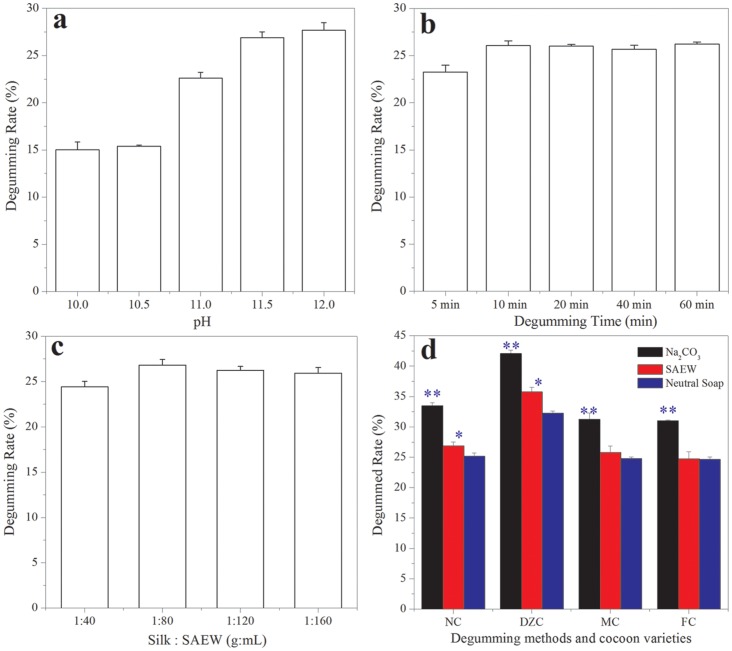
Effects of pH (a), degumming time (b), volume (c), methods and different strains of silkworm cocoon (d) on the degumming rate of silkworm cocoon. NC: white normal cocoon from the silkworm QingSong×HaoYue, (DZC): a yellow-green colored cocoon of the Daizo silkworm, MC and FC: both male and female cocoons are yellow and purple fluorescent under 365 nm UV light, respectively.

The silk degumming rate increased gradually with increased pH in SAEW as shown in Figure 2a. The degumming rate was 15% at pH 10.0 and pH 10.5; i.e. only the outer and middle layer sericin was removed from around the silk fibroin fiber. When the pH was increased to 11.0, the degumming rate rose to 23% and when the pH was increased to 11.50, the degumming rate increased to 26%, indicating the inner layer of sericin close to the silk fibroin was removed completely [30]. When the pH was increased to 12.00, the degumming rate increased very little, from 26 to 27%. In the preparation process, the generation of pH 12.0 SAEW was much slower than that of pH 11.5 SAEW; therefore, pH 11.5 SAEW was used in the subsequent experiments.

### Effect of Boiling Time on Silk Degumming Rate

The change of cocoon degumming rate as the boiling time in SAEW was increased from 5 min to 60 min as shown in Figure 2b. The degumming rate of boiling in SAEW for 5 min was 23% and this increased to 26% when the boiling time was increased to 10 min; increasing the boiling time to 20, 40 or 60 min had no further effect on the degumming rate, indicating that all of the sericin had been removed from around the fibroin fiber. Therefore, a boiling time of 20 min in pH 11.50 SAEW was used in the subsequent experiments.

### Effect of SAEW Volume on the Silk Degumming Rate

As shown in Figure 2c, the degumming rate was ∼25% for a cocoon shell to SAEW ratio of 1∶40 (W/V), which indicated that most or all of the sericin was removed from around the silk fibroin fiber. When the ratio was 1∶80, the degumming rate was increased to a maximum of nearly 27% but further increase of the ratio had no significant effect. These results show that the optimum ratio of cocoon shell to pH 11.50 SAEW for degumming is 1∶40∼80 (W/V) and the following experiments were all done at ratios within this range.

### Silk Degumming Rates of Different Strains of Silkworm Cocoon

Cocoons of silkworm strains NC, DZC, MC and FC were degummed by three different methods, neutral soap, pH 11.50 SAEW and Na_2_CO_3_ (described in Materials and methods) and the degumming rates are shown in Figure 2d. Boiling in 0.5% Na_2_CO_3_ for 30 min twice is the most widely used degumming method but it often causes a decline of the mechanical properties of the silk fiber, or even damage to the surface of the silk fibroin fiber, and the sericin protein chain is hydrolyzed into peptides and free amino acids. As shown in Figure 2d, the sericin content of silkworm cocoon shells varies significantly among strains. For example, the degumming rates were very high for the four strains of silkworm cocoons using the Na_2_CO_3_ method in comparison with neutral soap as control. These degumming rates have reached a significant difference (*p<0.01*) (Figure 2d). The degumming rates obtained by the neutral soap method were the lowest, ranging from 24.5 to 32.5% with the highest rate for DZC. The degumming rates obtained by the pH 11.50 SAEW method were a little higher than those obtained by the neutral soap method, ranging from 26 to 36% with the highest rate for DZC *(p<0.05).* These results show: (1) for the same degumming method, there were significant differences of sericin content in cocoon shells among silkworm strains; and (2) for the same strain of silkworm cocoon shells, the order of degumming rate achieved by different methods was: Na_2_CO_3_> SAEW>neutral soap. In general, (3) boiling in neutral soap causes the least damage to sericin in the silkworm cocoon shell; (4) the SAEW and the neutral soap degumming methods gave almost the same results. The degumming rates of cocoon shells from silkworm strains NC, (DZC), and FC and MC cocoons of the fluorescent strain were 27%, 36%, 26% and 25%, respectively. Recently, we developed a novel silk reeling method using SAEW as the swelling agent for silk fibers [31].

### Surface Property of Silk Fibroin Fiber

**Figure 3:**
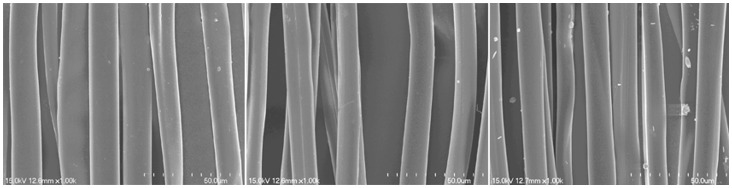
Surface images of single filament of silk fibroin degummed from different degumming methods. Degumming methods: Neutral soap (Left), pH11.50 SAEW (Middle), Sodium carbonate (Right).

The surface properties of silk fibroin fibers obtained by the Na_2_CO_3_, SAEW and neutral soap degumming methods observed in a scanning electron microscope (SEM; Hitachi S-4700 cold field emission microscope) at a magnification of 1000× are shown in Figure 3. These single filaments of silk fibroin are ∼10 µm in diameter but they are not standard cylindrical and their morphology is irregular. SEM observations showed that the surface of single filaments of the degummed silk fibroin was smooth and there was no evident difference among the products of the three degumming methods used in this study.

### Differential Scanning Calorimetry (DSC)

**Figure 4:**
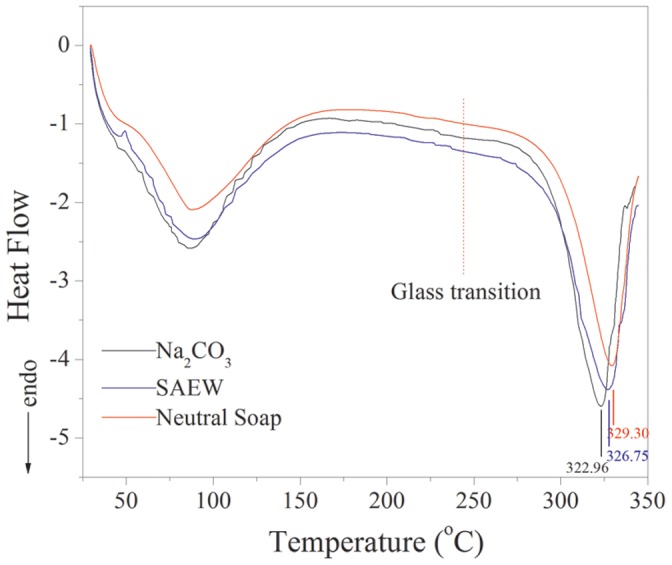
Effects of degumming methods on the thermal property of silk fibroin fiber.

The DSC curves of the silk fibroin fibers obtained by the Na_2_CO_3_, SAEW and neutral soap degumming methods are shown in Figure 4. The glass transition temperature was very similar for these silk fibroin fibers, indicating that these degumming methods had no significant impact on the fiber structure. However, the impact of these degumming methods on the thermal decomposition temperature of silk fibroin fibers was clear. The silk fiber degummed in neutral soap solution had the highest thermal decomposition temperature of 329.30°C, indicating this type of silk fiber has the greatest thermal stability. The thermal stability of fibers prepared by treatment with Na_2_CO_3_ was the least and the thermal decomposition temperature was 322.96°C, which is 6.5°C lower than that in the neutral soap group, indicating the silk fibroin fiber was altered to a considerable extent. The thermal stability of the silk fibroin fiber degummed in pH 11.50 SAEW was between those of the other treatments and its thermal decomposition temperature was 326.75°C. These results show the impact of treatment in pH 11.50 SAEW on the thermal properties of silk fibroin fiber was far less than that for Na_2_CO_3_ and a little higher than that for the neutral soap degumming methods.

### Tensile Properties of Single Filaments of Silk Fibroin

Table 2 gives the effects of three degumming methods on the tensile property of single filament of silk fibroin. First, the single filaments of silk fibroin from the neutral soap treatment had the greatest tensile strength; maximum load 5.83, cN, shift 4.29 mm, energy 0.17 mJ and tensile strain at maximum load 45.11%. The tensile properties of the single filaments from the pH 11.50 SAEW treatment were slightly (8%) lower than those of the neutral soap treatment; maximum load 5.39 cN, shift 3.96 mm, energy 0.15 mJ and tensile strain at maximum load 41.42%. The tensile properties of the fiber from the Na_2_CO_3_ treatment were much inferior; maximum load 3.70 cN, shift 2.42 mm, energy 0.07 mJ and tensile strain at maximum load 24.88%. Among these values, the energy and tensile strain at maximum load were decreased by ∼62% and ∼45% compared to those of the neutral soap group. The statistical analysis (Table 3) showed that there was(were) no significant difference(s) between the tensile properties of monofilaments of the silk fibroin derived from SAEW and the control group neutral soap; and there was(were) (a) very significant difference(s) (*P<0.01*) between it and those from the Na_2_CO_3_ treatment. These results suggest that the tensile properties of degummed silk fibroin fiber derived by treatment with pH 11.50 SAEW were very similar to those of the neutral soap treatment. Degumming or boiling in Na_2_CO_3_ results in a serious decrease of the tensile properties of the silk fibroin fiber and the results of the tensile test are entirely consistent with those obtained by DSC analysis.

**Table 2:** Tensile properties of single filament of silk fibroin.

Degumming methods	Sampling Position (group)	Maximum load(cN)±SD		Shift at the maximum load(mm)±SD		Energy at the maximum load (mJ)±SD		Tensile strain at the maximum load (%)±SD	
Neutral Soap	3	6.39620	1.05845	4.41242	0.20910	0.19425	0.04050	46.23617	1.73828
	4	5.91344	0.69787	4.47600	0.09331	0.18309	0.02430	46.96450	1.63587
	5	5.19388	0.53638	4.00562	0.21944	0.14442	0.02507	42.13454	1.85549
Means		5.83451	0.76423	4.29801	0.17395	0.17392	0.02996	45.11174	1.74321
SAEW	3	5.19492	1.75532	3.88897	0.16546	0.13972	0.04957	40.58424	1.94389
	4	6.16557	1.05471	4.18783	0.41294	0.18152	0.04642	44.02649	4.76703
	5	4.82880	0.56362	3.80838	0.53898	0.13677	0.01945	39.66198	5.95238
Means		5.39643	1.12455	3.96173	0.37246	0.15267	0.03848	41.42424	4.22110
Na_2_CO_3_	3	3.94465	0.80331	2.60415	0.62811	0.07737	0.03277	26.93301	6.68920
	4	3.76165	0.68551	2.45861	0.62249	0.06825	0.02553	25.14285	6.60920
	5	3.38846	0.74537	2.20702	0.16181	0.05436	0.01618	22.57305	1.56363
Means		3.69825**	0.74473	2.42326**	0.47080	0.06666**	0.02483	24.88297**	4.95401

Note: ten single filaments randomly taken among 100 single filaments (5 cm long) respectively in 3rd, 4th and 5th groups in each sample were measured and the above values are an average of 10 single filaments of silk fibroin.

SAEW and Na_2_CO_3_ two groups of test values in the table were analyzed by ANOVA with the control group (Neutral Soap). *p<0.05* means significant difference (*), and *p<0.01* means very significant difference (**).

**Table 3:** Statistical analysis of effects of degumming methods on tensile properties of single filament of silk fibroin by ANOVA.

Tensile properties	Source	DoF	Sum of Squares	Mean Square	F Value	P Value
Maximum load	Model	2	7.64	3.82	12.41	0.00738
	Error	6	1.85	0.31		
Shift at the maximum load	Model	2	5.99	3.00	61.82	0.00010
	Error	6	0.29	0.05		
Energy at the maximum load	Model	2	0.02	0.01	2.01	0.00219
	Error	6	0	0		
Tensile strain at the maximum load	Model	2	696.41	348.21	61.9	0.00010
	Error	6	33.75	5.62		

### Conclusions

1. The pH (11.50) of SAEW prepared from tap water by electrolysis was stable for one month in an airtight container. The hardness of SAEW was decreased by 30% compared to tap water and the K^+^ concentration was increased slightly (18%). The strong alkalinity of SAEW is mainly due to the high concentration of OH^–^ rather than a significant increase in alkaline ions produced by water electrolysis.
2. Silkworm cocoon shells were degummed thoroughly in pH 11.50 SAEW at a ratio of 1∶40∼80 (W/V) at 100°C for 20 min. The silk degumming rate of the SAEW method is slightly higher than or equal to that of the traditional neutral soap method. The tensile properties and thermal decomposition temperature of single filament of silk fibroin obtained by the SAEW method are almost the same as, or a little lower than, those of the neutral soap method. The boiling and scouring processing in the Na_2_CO_3_ method results in a serious decrease of tensile and thermal properties of the silk fibroin fiber.

## Materials and Methods

### Experimental Materials

The silkworm strains characterized by cocoons of different colors used in this study were the B. mori QingSong×HaoYue white, normal cocoon (NC) and the *B. mori* Daizo (DZC) yellow-green cocoon. The B. mori YingSu×YingXiao yellow male cocoon (MC) and the purple female cocoon (FC), which are fluorescent under 365 nm ultraviolet light [29], were kindly provided by Professor Xiao-Hua Yu, Sericulture Institute of Soochow University. The reasons for these varieties used in this experiment is due to the different content of silk sericin and other impurities in cocoon shell, indicating the broad applicability of SAEW as an new silk degumming agent.

### Preparation Principle and Equipment for the Production of Electrolyzed Water

The electrolysis of water is a chemical reaction. Water is a weak electrolyte within the ionization balance;
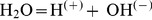
(1)

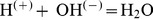
(2)

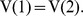
under 12 V (DC) hydrogen and oxygen release, hydrogen and hydroxide ions are constantly reduced, breaking the balance system, the water is kept ionized, V(1)>V(2), and the ongoing reaction is:



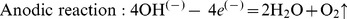


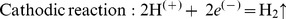


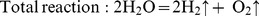



According to the above, the electrode reaction solutions are pH ∼7 at room temperature before or after electrolysis, but the pH value changes markedly at both ends of the electrodes in the electrolysis process: the OH^−^ concentration at the cathode is relatively increased due to H_2_ ↑ and pH increases; the H^+^ concentration at the anode is relatively increased because O_2_↑ and the pH decreases.

**Figure 5:**
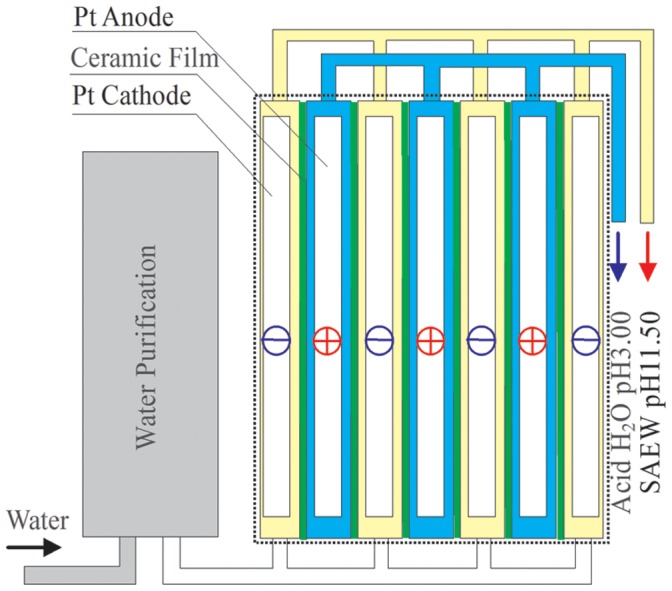
Structure diagram of SAEW electrolyzer.

This experiment used a laboratory-made electrolyzer composed of six groups of parallel electrolytic plates with a 12 V DC power supply for the electrolysis of tap water (Figure 5). The structure of the electrolyzer is shown in Figure 5.

### pH Stability of SAEW

Samples of pH 12.10 SAEW were stored under the following conditions: (1) in airtight closed containers at 4°C (4°C/closed) and (2) in open containers at 4°C (4°C/open). Samples of pH 11.60 SAEW were stored in (3) airtight closed containers at 25°C (25°C/closed) and (4) open containers at 25°C (25°C/open). Samples of acidic electrolyzed water (pH 3.00) were stored under the four sets of conditions described above for SAEW. The pH value of each storage type was measured daily for 30 days.

### Water Quality

Tap water (pH ∼8.00) was electrolyzed to produce a total of 18 L of pH 3.00 acidic electrolyzed water and 10 L of pH 11.50 SAEW using a laboratory-made electrolyzer. SAEW was filtered through a 0.45 µm paper filter and 0.7995 g (dry weight) of impurities was removed. Tap water from the same source was used to produce 10 L of ultrapure (18.25 MΩ.cm) water pH 8.23 (ULUPURE Co. Ltd., Shanghai, China). The tap water and the ultrapure water were used as controls. Water hardness, including calcium and magnesium ions, as well as K^+^ and Na^+^ were measured by an inductively coupled plasma atomic emission spectrometer (Vista MPX ICP, Varian USA). The four storage types of water described above were diluted 10-fold with the ultrapure water before analysis.

### Na_2_CO_3_ Degumming Method

A known weight of clean cocoon shells was immersed in 0.5% (W/V) Na_2_CO_3_ at a ratio of 1∶20 (W/V) and then heated for 30 min at 98°C. The resulting degummed silk was rinsed repeatedly (the washing water was kept) with 40°C deionized water then heated again for 30 min at 98°C in 0.5% Na_2_CO_3_. The degummed silk fibroin was washed 3 times (the washing water was kept) with ∼40°C deionized water to ensure complete removal of the sericin surrounding the silk fibroin fiber. After washing repeatedly with deionized water, the degummed silk fibroin fiber was air-dried at 105°C for 2 h and then weighed. The procedure was done in triplicate and the degumming rate was calculated as:
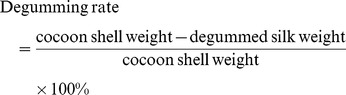



### Neutral Soap Degumming Method

A known weight of clean cocoon shells was added into a 0.2% (W/V) neutral soap solution at a ratio of 1∶100 (W/V) and heated at 96∼98°C for 30 min. The neutral soap solution was removed, replaced with a fresh solution and heated again five times. The degummed silk fibroin was washed with ∼40°C deionized water three times and these washings were saved and pooled. The degummed silk fibroin fiber was also washed repeatedly and air-dried at 105°C for 2 h. The procedure was done in triplicate and the degumming rate was calculated as described above.

### SAEW Degumming Method

pH 11.50 SAEW was used for degumming cocoons and calculation of the degumming rate. For each storage type of dry cocoon shells, a 2.00 g sample was mixed with pH 11.50 SAEW in a flask at a ratio of 1∶40 (W/V). The flask was placed into a boiling waterbath (100°C) for 20 min with constant stirring (120rpm). The degummed silk fiber from the SAEW solution was washed with double-distilled water and finally air-dried at 105°C for 2 h. The procedure was done in triplicate and the degumming rate was calculated as described above.

### Tensile Test of Single Filament of Degummed Silk Fibroin

**Figure 6:**
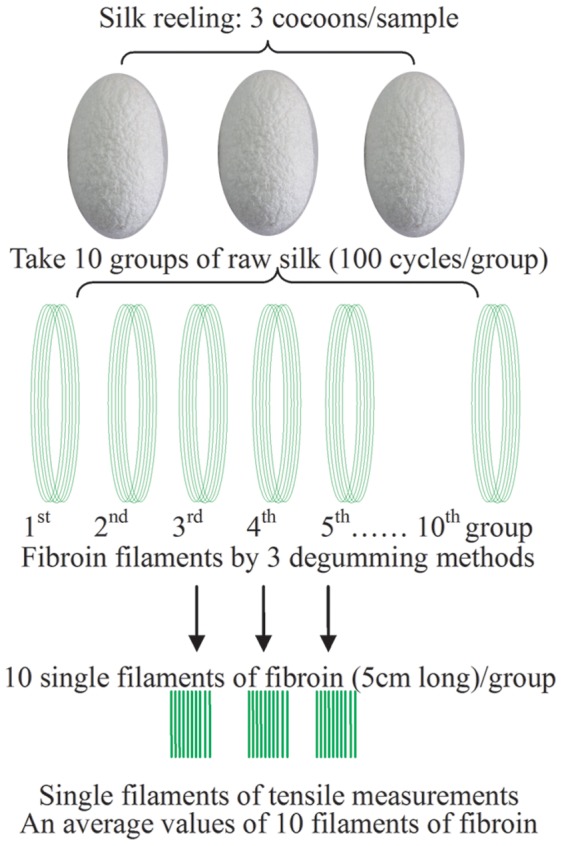
Reeling and sampling method for the measurement of single filament of fibroin.

The mature silkworm produces silk fibers >1000 meters long and the fineness of the fiber is not very uniform. In order to reduce error in the measurement of the tensile strength of a single filament of silk fibroin, each storage type of cooked cocoons was hand-reeled in water at 70°C (Figure 6). The hand-reeled silk fiber is often referred to as raw silk and is, in fact, a partly degummed silk fiber. The raw silks were degummed thoroughly by the three methods described above; neutral soap, SAEW and Na_2_CO_3_. Finally, the tensile properties of 50 mm long single filaments of silk fibroin were measured with a testing machine (INSTRON model 3365; Universal Testing Machine, INSTRON, US) to determine maximum load, shift at maximum load and energy at maximum load and the tensile strain at maximum load was calculated.
